# Circulating *Aspergillus fumigatus* DNA Is Quantitatively Correlated to Galactomannan in Serum

**DOI:** 10.3389/fmicb.2017.02040

**Published:** 2017-10-31

**Authors:** Alexandre Alanio, Jean Menotti, Maud Gits-Muselli, Samia Hamane, Blandine Denis, Emmanuel Rafoux, Régis Peffault de la Tour, Sophie Touratier, Anne Bergeron, Nicolas Guigue, Stéphane Bretagne

**Affiliations:** ^1^Laboratoire de Parasitologie-Mycologie, APHP, Paris, France; ^2^Université Paris-Diderot, Université Sorbonne Paris Cité, Paris, France; ^3^Unité de Mycologie Moléculaire, Institut Pasteur, CNRS URA 3012, Centre National de Référence des Mycoses Invasives et des Antifongiques, URA 3012, Paris, France; ^4^Service de Maladie Infectieuses et Tropicales, AP-HP, Paris, France; ^5^Service d’Hématologie Adulte, AP-HP, Paris, France; ^6^Service d’Hématologie-Greffe de Moelle, APHP, Paris, France; ^7^Service de Pharmacie, APHP, Paris, France; ^8^Service de Pneumologie, APHP, Paris, France

**Keywords:** *Aspergillus fumigatus*, invasive aspergillosis, galactomannan, quantitative real-time PCR, circulating DNA

## Abstract

The performance of antigen galactomannan (GM) for diagnosing invasive aspergillosis (IA) is hampered by the occurrence of false-positive results. Quantitative PCR has been proposed to improve the diagnosis of IA. Therefore, we analyzed the value of performing a PCR test to the GM-positive serum sample. Using a quantitative PCR assay specific for *Aspergillus fumigatus* 28S ribosomal DNA, we retrospectively tested 422 GM-positive (Platelia Bio-Rad kit) serum samples collected over 1 year from 147 patients. The cases were classified based on EORTC criteria as “proven,” “probable,” and “no–IA” before availability of the PCR results. After exclusion of 65 samples for non-reproducibility of GM positivity (*n* = 62) or PCR inhibition (*n* = 3), 75 (21.0%) of the remaining 357 samples were PCR-positive. GM and fungal DNA showed a significantly positive correlation (*p* < 0.0001, *R*^2^ = 0.27, slope = 0.98 ± 0.19). At least one PCR-positive result was observed in 63.3% (31/49) of IA patients and in 13.2% (13/98) of non-IA patients (*p* < 0.0001). The PCR positivity was also associated with the presence of other microbiological criteria among the 44 patients with IA and complete mycological workup (*p* = 0.014), as well as a higher mortality rate at six months among the 135 patients with hematological conditions (*p* = 0.0198). Overall, we found a quantitative correlation between serum GM and circulating DNA with an increased likelihood of IA when both were positive. A PCR-positive result also supported a higher fungal load when GM was already positive. We advocate adding a PCR test for every confirmed GM-positive serum sample.

## Introduction

The diagnosis of probable and proven invasive aspergillosis (IA) requires microbiological criteria, which include the galactomannan (GM) antigen (De Pauw et al., 2008). This antigen is produced by several molds including *Aspergillus fumigatus*, the main species responsible for IA (Lortholary et al., 2011). For serum, the test is mainly used as a screening test to initiate a diagnostic workup or to start antifungal therapy as soon as possible (Marchetti et al., 2012). However, concern has always been raised about the rate of false positivity using the Platelia Aspergillus Ag assay (Bio-Rad Laboratories, Marnes la Coquette, France) (Marchetti et al., 2012). We recently showed the importance of excluding unreproducible positive results by testing GM-positive samples twice (Guigue et al., 2015). However, numerous GM-positive samples cannot be ascribed to IA even after an intensive diagnostic work-up including imaging and mycology with direct examination and culture. At a cut-off value 0.5 ODI and an IA prevalence of 8%, a meta-analysis showed 19% of false positives (Leeflang et al., 2008).

Real-time quantitative PCR assays have been proposed to improve the diagnosis of IA. With the advent of PCR, several technical procedures have been recommended for testing serum (White et al., 2011). With such improvements, PCR should be recommended for diagnosing IA (White et al., 2015). The combination of both tests to improve the clinical utility for the diagnosis of IA has been advocated for many years (Bretagne et al., 1998; Barnes et al., 2013). In a recent meta-analysis, the association of positive results for both tests was highly suggestive of an active infection with a positive predictive value of 88% (Arvanitis et al., 2015). However, a screening strategy utilizing twice weekly GM and PCR testing is questioned with the generalization of anti-mold prophylaxis (Patterson et al., 2016). In decreasing the prevalence of IA, the efficiency of the screening strategy decreases (Leeflang et al., 2008). In these conditions, GM testing is integrated in a diagnostic work-up without the previous serial tests to interpret the results. Thus, a positive GM results should be interpreted on a single result. One possibility is to ask for a second serum sample. However, this can delay the initiation of an appropriate therapy. We wondered whether adding a PCR test for every GM-positive sample without waiting for additional samples could improve the diagnosis of IA.

## Materials and Methods

### Ethics Statement

The present study was a non-interventional retrospective study performed using biological material and clinical data obtained for standard diagnostics without any supplementary sampling and no change in the usual procedures. French Public Health Law (CSP Art L1121-1.1) does not require specific approval from an ethics committee for this study which is exempted from specific informed consent application.

### Serum Samples Collection and GM Detection

From January 1st, 2013 to December 31st, 2013, 7628 serum samples were tested using the Platelia Bio-Rad kit mainly as part of the screening of 1374 patients at risk of IA according to previous recommendations (Marchetti et al., 2012). GM detection was performed according to the manufacturer’s instructions. The results were inferred from the ratio of the optical density (OD) results from the sample and the controls and are expressed as GM-OD index (GM-ODI). For each positive serum sample (GM-ODI > 0.5), another test on the same serum sample was performed the next day as part of our routine practice (Guigue et al., 2015). Only the samples that tested positive twice were considered as positive, and the mean of the two GM-ODIs was used for further analyses. The serum samples that turned negative were considered as unreproducible results (Guigue et al., 2015). The serum samples were stored at -80°C until further analysis.

### DNA Detection

All serum samples tested positive with more than 1 ml available were analyzed. Storage at -80°C before PCR did not exceed 2 years. After thawing, DNA from 1 mL of serum was extracted using the Qiasymphony DSP virus/Pathogen Mini kit (Qiagen) and a Qiasymphony apparatus (Qiagen), eluted in 85 μL, and tested in duplicate using the 28S rDNA PCR assay previously reported (Challier et al., 2004). Primer and probe concentrations were set at 0.3 and 0.1 μM in the 480 probe Master (Roche), respectively, and the PCR assay was performed in a LightCycler 480 instrument (Roche).

The results were expressed in quantification cycles (*C*q), with higher values indicating less targeted DNA in the sample. Positivity was defined by at least one of the two duplicates having *C*q ≤ 45 cycles. The mean value of the duplicates was retained for further comparisons when both were positive and the single value when one replicate was positive alone. DNA extraction and amplification yields were assessed using the Simplexa Extraction and Amplification Control Set (Focus Diagnostics, Cypress, CA, United States) as an internal control (IC). The PCR assay was performed blind to interpretation of the GM results (true or false positives) and to the clinical classification.

### Mycology Laboratory Result

Respiratory specimens [bronchoalveolar lavage (BAL) fluid, induced sputum, sputum] were split into two parts. One part underwent direct examination using microscopy after staining with calcofluor (BD Biosciences) in KOH (10%). The other part was seeded on Sabouraud dextrose agar with gentamycin and chloramphenicol (Bio-Rad) and incubated at 30 and 37°C. Every positive culture was identified using phenotypic methods. Molecular identification was done based on sequencing three different loci (Internal Transcribed Spacer, beta-tubulin, calmodulin). Sequences were then posted in the Mycobank database^1^ and Institut Pasteur FungiBank^2^.

### Patient Classification

Every four months, a local multidisciplinary medical committee analyses each effective anti-mold therapy recorded in the pharmacy department and classifies the patients as ‘proven,’ ‘probable,’ IA, or ‘no-IA’ according to criteria from the European Organization for Research and Treatment of Cancer and from the Invasive Fungal Infections Cooperative Group and the National Institute of Allergy and Infectious Diseases Mycoses Study Group (EORTC/MSG) (De Pauw et al., 2008). For patients without hematological condition or solid organ transplantation not responding to the EORTC/MSG definition, *ad hoc* classification was consensually performed by the local committee. The patient outcome was censored at 6 months (183 days).

### Graphs and Statistical Analysis

Chi-2 test and Fisher’s exact test were used for contingency tables analyses for calculation of statistical association. The potential relationship between GM and *C*q was studied using linear regression and for comparison of slopes, a *P*-value (two-tailed) testing the null hypothesis that the slopes are all identical (the lines are parallel) was calculated. Survivals were determined by Kaplan–Meier method and compared by the Mantel–Cox test. For comparisons of quantitative data, we performed unpaired two-tailed *t*-test for normally distributed data. *P*-values of <0.05 were considered significant. All analyses and graphs were performed using Prism software v.6.0 (Graphpad).

## Results

Of the 422 GM-positive serum samples tested by PCR, three were excluded because of IC amplification failure. Among the 419 remaining samples, 62 were unreproducible GM-positive samples, i.e., the first positive result (median ODI: 0.75, interquartile range: 0.55–1.005) tested negative upon retesting. All were PCR-negative. These 62 samples were from 53 patients (median number 1; interquartile range 1–1 range 1–3). None of these patients developed IA.

**FIGURE 1:**
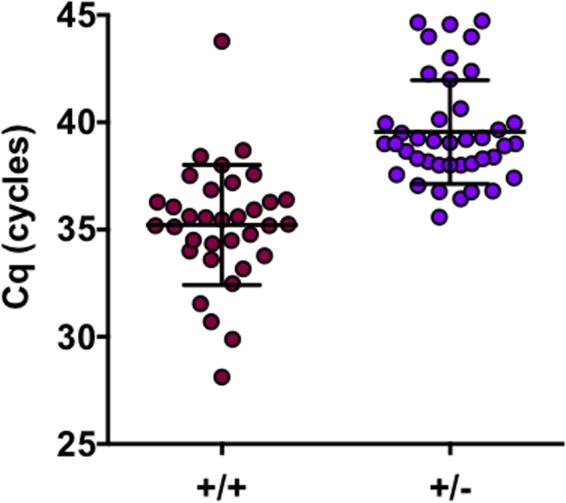
Results of the duplicates performed for each PCR-positive sample. The positive duplicates (+/+) had lower mean *C*q values than the sample (+/–) with only one duplicate positive (*t*-test; *p* < 0.001).

**FIGURE 2:**
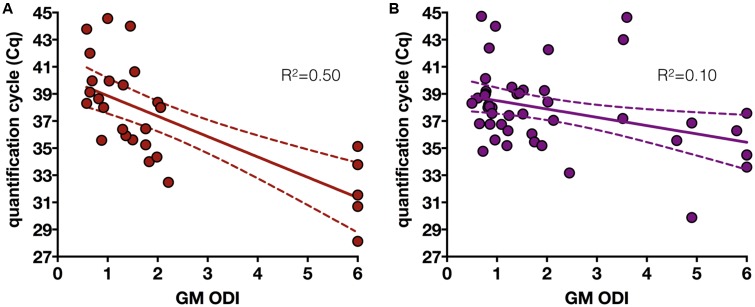
Correlation between galactomannan index (GM-ODI) and quantification cycle (*C*q) of *Aspergillus fumigatus* PCR assay in GM-positive serum samples obtained before **(A)** or after **(B)** initiation of antifungal therapy.

The remaining 357 samples were confirmed to be true GM-positive samples after retesting (median difference: ODI 0.11 interquartile range [0.04–0.22], mean ± SD 0.19 ± 0.27). Among these, 75 (21.0%) were PCR-positive with a median of one positive sample per patient [interquartile range 1–2; range 1–13]. For the duplicates, they were more consistently both positive when the *C*q was below 38 (**Figure 1**). When looking at quantitative values of the assays, the GM-ODI and the *C*q showed an inversely significant correlation (slope = -0.98 ± 0.19, *R*^2^ = 0.27; *p* < 0.0001). When considering PCR results from samples collected before or during antifungal therapy, the correlation was significantly improved (*p* = 0.019) for the samples before therapy (*n* = 28, slope = -1.5 ± 0.29, *R*^2^ = 0.50; *p* < 0.0001) compared to those collected after the initiation of antifungal treatment (*n* = 47, slope = -0.61 ± 0.23, *R*^2^ = 0.13; *p* = 0.01) (**Figure 2**).

The 357 GM-positive samples (median 1; range 1–17 samples per patient) were from 147 patients with mainly hematological diseases as an underlying condition (**Table 1**), of whom 49 (33.4%) were classified as having IA (47 with probable IA, 2 with proven IA) and the remaining 98 (66.7%) as not having probable or proven IA (**Table 1**). At least one PCR-positive serum sample was observed in 31 (63.3%) of the 49 patients with IA, including the two proven cases, and in 13 (13.2%) of the 98 patients without IA (*p* < 0.0001) (**Table 1**). Notably, none of the 10 patients with common variable immunodeficiency disease (CIVD) were considered as having IA and all were PCR-negative (**Table 1**).

**Table 1:** Underlying disease or risk factors of invasive fungal diseases of the 147 adults patients with the PCR results of the 357 true GM-positive samples tested, and the results according to the final diagnosis of invasive aspergillosis (IA).

Underlying diseases or condition	Number of patients (*n* = 147)	Number of samples (*n* = 357)	Number of PCR samples (*n* = 75)	Number of IA patients (*n* = 49)	Number of PCR-positive IA patients (*n* = 31)	Number of treated patients wo IA diagnosis (*n* = 18)
Myeloid disorders	26 (17.7)	49 [1–6]	14 (28.6)	7 (26.9)	5 (71.4)	5 (28)
Acute myeloid leukemia	22 (15)	39 [1–5]	9 (23.1)	4 (18.2)	4 (100)	3 (17)
Myelodysplasia	4 (2.7)	10 [1–6]	5 (50)	3 (75)	1 (33.3)	2 (11)
Acute lymphoid leukemia	13 (8.8)	26 [1–6]	5 (19.2)	3 (23.1)	2 (66.7)	5 (28)
Chronic lymphoproliferative disorders	39 (26.5)	76 [1–7]	18 (23.7)	22 (56.4)	11 (50)	4 (23)
Lymphoma	26 (17.7)	50 [1–7]	11 (22)	14 (53.8)	7 (50)	3 (17)
Multiple myeloma	11 (7.5)	21 [1–7]	6 (28.6)	6 (54.5)	3 (50)	1 (6)
Chronic lymphoid leukemia	2 (1.4)	5 [1–4]	1 (20)	2 (100)	1 (50)	0 (0)
Allogeneic stem cell transplantation^a^	42 (28.6)	153 [1–17]	29 (19)	11 (26.2)	8 (72.7)	2 (11)
Renal transplantation	6 (4.1)	15 [1–5]	6 (40)	3 (50)	2 (66.7)	1 (6)
CVID^b^	10 (6.8)	17 [1–5]	0 (0)	0 (0)	0 (0)	1 (6)
AIDS	6 (4.1)	8 [1–3]	1 (12.5)	1 (16.7)	1 (100)	0 (0)
Others^c^	5 (3.4)	13 [1–5]	2 (15.4)	2 (40)	2 (100)	0 (0)


*Numbers in () represent % and numbers in [] represent range/patient; wo, without*.

^a^*Underlying diseases include myeloid disorders (*n* = 18), acute lymphoid leukemia (*n* = 7), chronic lymphoproliferative disorders (*n* = 16), and sickle cell disease (*n* = 1)*.

^b^*Common variable immunodeficiency disease*.

^c^*Includes: pulmonary carcinoma (*n* = 2); amyloidosis (*n* = 1); Still disease (*n* = 1); Intensive care unit (*n* = 1)*.

*The last column represents the PCR-positive patients who received effective anti-mold therapy despite the absence of all the EORTC/MSG criteria for diagnosing IA*.

Among the 18 patients with IA who were PCR-negative, one patient (five samples) had probable IA due to *Emericella quadrilineata*, and one patient (one sample) had probable IA due to *A. flavus*, based on culture results. Both species were not detected by our PCR assay. Among the 98 patients without IA who were GM-positive, one (one sample) had cryptococcosis and one (three samples) had histoplasmosis. Among the remaining 96 patients, who were GM-positive, 18 (18.75%) were given effective anti-mold therapy and 12 (66.7%) of them were PCR-positive, in contrast with only one (1.3%) PCR-positive patient among the 77 GM-positive patients who were not given effective anti-mold therapy (*p* < 0.0001) (**Table 1**).

Among the 49 patients with IA, 44 had other investigations performed (direct microscopic examination, culture, BAL GM testing). Among these 44 patients, the percentage of PCR-positive patients increased with the number of other positive criteria, from 36.8% (7/19) with GM positivity alone to 76.9% (19/25) when GM, culture and/or BAL GM was positive (*p* = 0.014) (**Table 2**).

**Table 2:** Comparison between PCR-positive results and the presence of other positive microbiological investigation [direct microscopy, culture, and bronchoalveolar lavage (BAL) GM testing] in 44 patients with probable or proven invasive aspergillosis and at least one galactomannan (GM) positive sample.

Results of mycological investigations	PCR-positive patients (*n* = 26) (%)	PCR-negative patients (*n* = 18) (%)	*P*-value
Serum GM positivity alone	7 (36.8)	12 (63.2)	0.014
Serum GM positivity associated with other microbiological criteria	19 (76.0)	6 (24.0)	


**FIGURE 3:**
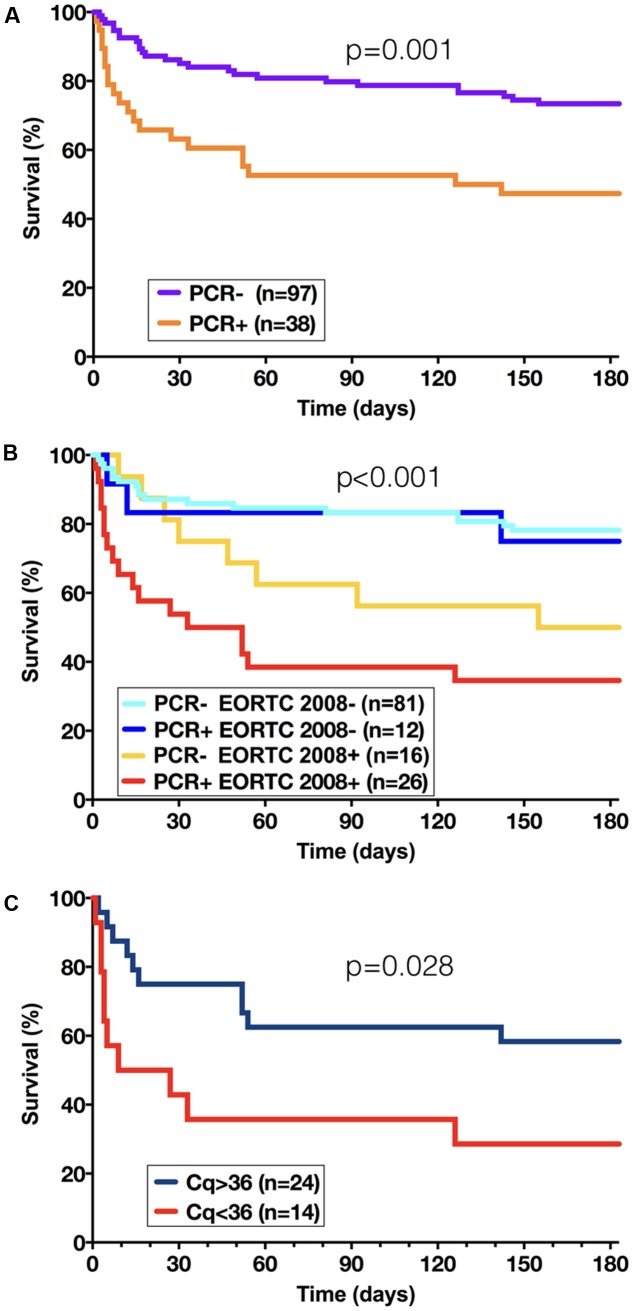
Survival curves according to the presence or not of a PCR-positive result on GM-positive samples **(A)**, to the presence or not of a PCR-positive result associated with EORTC/MSG criteria for IA diagnosis **(B)**, and according to the circulating fungal load as expressed as a *C*q value **(C)**.

We also analyzed the survival of the patients for whom a 6-month follow up was available (*n* = 135) and who had underlying hematological diseases or allogeneic hematopoietic stem cell transplantation to avoid confusion with the other underlying diseases (**Figures 3A–C**). Survival was significantly lower (*p* = 0.001) in PCR-positive patients compared to PCR-negative patients (**Figure 3A**). We further analyzed the data according to the presence or not of the EORTC/MSG 2008 criteria (**Figure 3B**). The patients without EORTC/MSG criteria had a better survival (*p* < 0.001) whatever the PCR result than patients who fulfilled the EORTC/MSG criteria who exhibited the worst prognosis when PCR was positive, even if the difference between PCR-negative and PCR-positive patients did not reach significance (*p* = 0.073). When analyzing the quantitative results (*n* = 38 patients), the patients with a *C*q < 36 on the first PCR-positive sample had a shorter survival than patient with a *C*q > 36 (*p* = 0.028) (**Figure 3C**). The *C*q values of the first PCR-positive sample were significantly lower (*t*-test, *p* = 0.0066) in the deceased patients (mean ± SEM = 34.8 ± 1.2) than in the patients alive at week 2 (mean ± SEM = 38.6 ± 1.3).

## Discussion

The aim of the present study was to improve the specificity of the GM test by adding on the same tube the detection of circulating *A. fumigatus* DNA. We found not only an association between PCR positivity and the presence of IA, but also for the first time a quantitative correlation between GM and PCR results in serum. Moreover, all our results suggest that a PCR-positive result once GM is already positive is associated with a poorer prognosis.

Based on the EORTC/MSG classification performed blind to the PCR results, the percentage of patients with at least one PCR-positive result increased from 13.2% in the non-IA group to 63.2% in the IA group (*p* < 0.0001). However, our results clearly show that the EORCT/MSG classification does not always fit with the clinical decision underlining the issue of the GM assay specificity (Leeflang et al., 2008; Marchetti et al., 2012). Indeed, among the GM-positive patients, only 18.75% were prescribed effective anti-mold therapy. Interestingly, 66.7% of the patients who received antifungal therapy were PCR-positive, and this result was not known when the clinical decision was made. Therefore, PCR performed when GM is already positive can improve the specificity of the GM result.

We also report for the first time that fungal DNA and GM titers are positively correlated. In parallel, we observed a higher mortality at 6 months in hematology when the patients were PCR-positive. Our hypothesis is that PCR positivity indicates a more advanced stage of the IA, at least when GM is already positive. This is also supported by the significant association of PCR positivity with other microbiological criteria. This finding is consistent with the poorer prognosis reported when several mycological criteria are present (Lortholary et al., 2011). Therefore, if PCR positivity increases the specificity of GM, this positivity is also associated with a higher fungal load, and thus, with a poorer prognosis. This finding is also coherent with the poor prognosis observed when the GM titers remain high (Boutboul et al., 2002; Nouér et al., 2011; Bergeron et al., 2012).

Although PCR could improve the specificity of some GM-positive serum samples, many GM-positive samples remain PCR negative (36.7%), even after the exclusion of unreproducible GM results (Guigue et al., 2015). We have already observed more GM-positive than PCR-positive serum samples in different populations (Bretagne et al., 1998; Millon et al., 2005; Botterel et al., 2008). Similar findings have been recently reported (Aguado et al., 2015; Imbert et al., 2016). For instance, less than 24% of GM-positive patients had at least one PCR-positive serum sample in a randomized trial (Aguado et al., 2015). A few of these GM-positive PCR-negative samples could be related to cross-reactions during infections by other fungi such as cryptococcosis (Dalle et al., 2005) or histoplasmosis (Rivière et al., 2012). They can also be due to *Aspergillus* species not targeted by the present primers specific for *A. fumigatus* (Challier et al., 2004), which represent a very small number of patients given the high predominance of *A. fumigatus* among the species responsible for IA in our patient population (Lortholary et al., 2011). GM contamination of transfused products and specially fungus-derived antibiotics are also always a concern, although to a lesser extend nowadays for piperacillin-tazobactam (Vergidis et al., 2014).

The main explanation for GM-positive PCR-negative results is probably the different kinetics of the two biomarkers. *In vitro* studies have shown that the maximum release of GM and *A. fumigatus* DNA was correlated with increased biomass during culture, with GM being detectable earlier than DNA (Mennink-Kersten et al., 2006; Morton et al., 2010). Several animal models of pulmonary aspergillosis also showed that GM is detected earlier, usually 24–48 h before DNA^.^(Becker et al., 2000; Ahmad et al., 2014; Lin et al., 2014). Using droplet digital PCR, we recently showed that the circulating *A. fumigatus* DNA detected in patients is fragmented, and we hypothesized that such DNA comes from dying hyphae, is extracellular and circulates in the serum (Alanio et al., 2016). Indeed, extracellular DNA has been detected increasingly over time in a biofilm model of *A. fumigatus* and was shown to be produced through autolysis (Rajendran et al., 2013). Besides the differences of kinetics between the two markers during an active IA, other differences may occur. We observed a lower correlation between GM and DNA when antifungals are given suggesting a different impact of treatment on the release of biomarkers in the bloodstream, which warrants further investigations. This suggests that DNA could be a poorer marker for following the efficiency of therapy compared to serum GM (Boutboul et al., 2002; Nouér et al., 2011; Bergeron et al., 2012). Another reason for discrepancies between GM titers and PCR *C*t may be related to the infecting isolates. Indeed, possible differences in the copy numbers of the rRNA repeats (between 61 and 86 copies/genome) chosen as the target for amplification could affect quantification through PCR according to the infecting isolate (Alanio et al., 2016).

Our study was not designed to address the issue of a PCR-positive signal before the onset of GM positivity. In guinea pigs infected by inhalation, circulating GM antigen was above the threshold of detection at day 3 and steadily rose thereafter whereas the PCR assay detected 10 conidial equivalents as soon as one hour, with a peak of 20 conidial equivalents at 24 h post infection (Vallor et al., 2008). This observation raises the issue of the detection of circulating conidia, which are probably engulfed in macrophages after a pulmonary challenge, either directly or through dendritic cells as reported for *Histoplasma capsulatum* (Lin et al., 2005) and *Cryptococcus neoformans* (Lortholary et al., 1999). Aguado et al. (2015) reported PCR-positive results before GM positivity in 42% of the 30 patients analyzed, while GM before PCR positivity occurred in 38%. We have also reported PCR positivity before GM positivity and concluded that a PCR-positive result accelerates the early detection of IA independently of the other diagnostic information (Schwarzinger et al., 2013). Additional specific studies are needed to determine whether PCR positivity before or after GM positivity could respond to different pathology processes (detection of fungal elements vs. circulating DNA). These studies should nevertheless be difficult to implement given the generalization of anti-mold prophylaxis. Indeed, some authors suggest that the potential of PCR to play a decisive role in the diagnosis and management of IA should be restricted to centers not applying primary antifungal mold prophylaxis (Springer et al., 2016).

We acknowledge some limitations to our study due to the retrospective design. The association between more microbiological criteria and PCR positivity might be a consequence of more investigations in patients with a higher suspicion index. Similarly, the higher mortality observed in PCR patients with hematological conditions does not mean that the patients died of IA in light of the difficulties in assessing the prognosis of IA (Segal et al., 2008). For the PCR protocol, several criticisms can be addressed. We decided to consider PCR-positive samples as having a positive threshold of *C*q ≤ 45, and we considered a sample positive even when the duplicate was not positive. A lower *C*q value is often used for censoring the results when a risk of unspecific positivity is suspected. For instance, Johnson et al. censored their results at 40 cycles when using primers and a probe not specifically designed for *A. fumigatus* (Johnson et al., 2012). If a threshold of 40 had been used in our study, 12 samples from 10 patients would have become PCR negative, restricting the interest of the PCR to very limited patients with a high fungal DNA load. However, there is no consensus on the threshold to be used even if ROC analysis of results of a multicenter study indicated a good diagnostic accuracy of a *C*q ≤ 43 cycles (White et al., 2011). Six patients of the present study had *C*q between ≥43 and ≤45 cycles. One out these six had a probable IA with *A. fumigatus* positive culture from respiratory specimens. Thus, despite the difficulty to obtain positive duplicates when dealing with low fungal loads (e.g., when *C*q > 38) because it may be normal to obtain negative results of replicates according to the Poisson’s law (Alanio and Bretagne, 2014), we think a *C*q ≤ 45 should be taken into account for diagnosing IA for a given patient. The other possibility is to wait for additional samples and expect a higher fungal load on the subsequent results to confirm the diagnosis but this attitude could be deleterious for the patient. Because no systematic screening of other samples was part of our study restricted to samples known to be GM-positive, we cannot comment the benefit to wait for additional samples to improve the specificity of the PCR results. One can also criticize our choice to focus on *A. fumigatus* in light of the increase of other *Aspergillus* species in IA (Lionakis et al., 2005) or mixed infections (de Fontbrune et al., 2014). However, restriction to a specific species decreases the risk of PCR positivity from environmental non-*fumigatus* DNA, especially if high *C*q are to be considered (Alanio and Bretagne, 2014), although contamination with *A. fumigatus* DNA cannot be completely excluded (Harrison et al., 2010; Millon et al., 2010). Moreover, since all the *Aspergillus* species do not equally produced GM in the same quantity (Xavier et al., 2013), quantitative comparison between GM and circulating DNA would have been biased if a non-*A. fumigatus* specific PCR had been used.

## Conclusion

The diagnosis of IA is a dynamic process where all the diagnostic elements are not obtained simultaneously. Since prospective screening is questioned with the generalization of antifungal prophylaxis in patients at risk of IA, resulting in a decrease in GM performance (Patterson et al., 2016), GM should become the first test requested by clinicians in case of febrile pneumonia for patients at risk of invasive mold infection. Our results clearly show the added value of PCR tests for every GM-positive serum sample to increase the probability of IA diagnosis, without waiting for additional samples. The two markers provide parallel quantitative information in accordance with the fungal load. A PCR-positive result is associated with a poorer outcome, probably as the witness of a higher fungal load, in accordance with the positivity of the other microbiological investigations.

## Author Contributions

AA and SB designed the project, performed the analyses, and wrote the manuscript. JM, MG-M, SH, and NG collected and analyzed the laboratory experiments. BD, ER, RPdT, ST, and AB classified the patients based on medical file analysis. All authors read, commented, and approved the final manuscript.

## Conflict of Interest Statement

The authors declare that the research was conducted in the absence of any commercial or financial relationships that could be construed as a potential conflict of interest.

## Footnotes

1 http://www.mycobank.org/BioloMICSSequences.aspx?expandparm=f&file=all

2 http://fungibank.pasteur.fr/
